# Schlafen4^+^-MDSC in *Helicobacter*-induced gastric metaplasia reveals role for GTPases

**DOI:** 10.3389/fimmu.2023.1139391

**Published:** 2023-06-02

**Authors:** Lin Ding, Sulaiman Sheriff, Ricky A. Sontz, Juanita L. Merchant

**Affiliations:** Department of Medicine-Gastroenterology, University of Arizona, Tucson, AZ, United States

**Keywords:** tumor microenvironment, single cell-RNA sequencing, GTPase, cGMP, sildenafil

## Abstract

**Introduction:**

MDSCs express SCHLAFEN 4 (SLFN4) in *Helicobacter*-infected stomachs coincident with spasmolytic polypeptide-expressing metaplasia (SPEM), a precursor of gastric cancer. We aimed to characterize SLFN4^+^ cell identity and the role of Slfn4 in these cells.

**Methods:**

Single-cell RNA sequencing was performed on immune cells sorted from PBMCs and stomachs prepared from uninfected and 6-month *H. felis*-infected mice. Knockdown of Slfn4 by siRNA or PDE5/6 inhibition by sildenafil were performed in vitro. Intracellular ATP/GTP levels and GTPase activity of immunoprecipitated *Slfn4* complexes were measured using the GTPase-Glo assay kit. The intracellular level of ROS was quantified by the DCF-DA fluorescent staining, and apoptosis was determined by cleaved Caspase-3 and Annexin V expression. *Gli1CreERT2 x Slfn4* ^fl/fl^ mice were generated and infected with *H. felis*. Sildenafil was administered twice over 2 weeks by gavaging *H. felis* infected mice ~4 months after inoculation once SPEM had developed.

**Results:**

*Slfn4* was highly induced in both monocytic and granulocytic MDSCs from infected stomachs. Both *Slfn4* ^+^-MDSC populations exhibited strong transcriptional signatures for type-I interferon responsive GTPases and exhibited T cell suppressor function. SLFN4-containing protein complexes immunoprecipitated from myeloid cell cultures treated with IFNa exhibited GTPase activity. Knocking down Slfn4 or PDE5/6 inhibition with sildenafil blocked IFNa induction of GTP, SLFN4 and NOS2. Moreover, IFNa induction of *Slfn* ^+^-MDSC function was inhibited by inducing their reactive oxygen species (ROS) production and apoptosis through protein kinase G activation. Accordingly, in vivo disruption of Slfn4 in *Gli1CreERT2 x Slfn4* ^fl/fl^ mice or pharmacologic inhibition by sildenafil after Helicobacter infection also suppressed SLFN4 and NOS2, reversed T cell suppression and mitigated SPEM development.

**Conclusion:**

Taken together, SLFN4 regulates the activity of the GTPase pathway in MDSCs and precludes these cells from succumbing to the massive ROS generation when they acquire MDSC function.

## Introduction

Schlafens (SLFN) are a novel and poorly understood family of proteins implicated in myeloid cell differentiation, proliferation, and immune responses (1, 2). Others and we previously reported that mouse SLFN4 and its human ortholog SLFN12L mark a group of cells that migrate to the stomach during a *Helicobacter* infection and subsequently acquire myeloid derived suppressor cell (MDSC) function coincident with the emergence of gastric intestinal metaplasia and spasmolytic polypeptide-expressing metaplasia (SPEM) or collectively gastric metaplasia (3, 4). In humans, the appearance of SLFN12L correlates with gastric metaplasia and gastric adenocarcinoma (GAC) (3, 4).

We propose that strategies to eliminate this MDSC subset may affect the development of gastric metaplasia and ostensibly gastric adenocarcinoma. However, we have limited understanding of SLFN4^+^ cell identity and function aside from their T cell suppressor activity. Prior bulk RNA-Seq analysis of tdTomato-labeled *Slfn4^+^*-MDSCs from infected mouse stomachs indicate that there is a >2000-fold increase in *Nos2* expression (3), and increased expression of several IFNα-inducible GTPases, such as guanylate binding proteins (GBPs), IIGP1 and GVIN, which hydrolyze guanine triphosphate (GTP) to guanosine diphosphate (GDP) and provide energy for cellular activities (5). MDSCs use arginase-1 (ARG1) and inducible nitric oxide synthase (NOS2), key enzymes in L-arginine catabolism, which blunt T cell-mediated antitumor immunity either individually or together (6, 7). The process of GTP metabolism is an important regulatory mechanism that controls the activity of NOS2 (8). NOS2 catalyzes the oxidation of L-arginine to nitric oxide (NO), a co-factor for soluble guanylate cyclase (sGC), which converts GTP to cGMP. cGMP-dependent phosphodiesterases (PDEs) hydrolyze cGMP to GMP. Subsequently, a collection of enzymes including guanylate kinases (GUK), nucleoside diphosphate kinases and GTPases, convert GMP back to GTP to regenerate the substrate that fuels the NO-sGC-cGMP pathway. Recently, three-dimensional modeling revealed that the C-terminus of the SLFN12 protein contains a GTPase-like domain, analogous to the dynamin-like family of large GTPases, e.g., dynamin, OPA1, GBPs and MX proteins (9). The GTPase domain is found in all group II-III SLFN proteins, including murine SLFN4 and its human ortholog SLFN12L (10). Therefore, the SLFN family may itself possess GTPase activity or form a complex with other GTPases.

In the current study, we performed single-cell RNA sequencing (scRNA-seq) analysis on the immune cell populations sorted from either (peripheral blood monocytes (PBMCs) or the stomachs of *H. felis*-infected mice to characterize SLFN4-expressing immune populations. Although *Slfn4+* cells occurred in a small number of myeloid cells of the uninfected stomach, its expression in both M-MDSCs and G-MDSCs dramatically increased 6 months after *H. felis* infection whereas, genetic deletion of the *Slfn4* locus or pharmacologic inhibition of PDE5,6 activity with sildenafil suppressed MDSC-mediated T cell suppression and mitigated metaplastic development, suggesting an important role for these SLFN4-expressing cells in the pathogenesis of gastric metaplasia and eventually gastric cancer.

## Materials and methods

### Transgenic mice

To determine if *Slfn4* is required for the MDSC function and metaplastic development, a *Slfn4^FLEx6FL^* was created after purchasing embryos carrying the *Slfn4^tm1a(EUCOMM)Wtsi^* mouse on a C57BL/6N background (MO-146694129) from the Mutant Mouse Regional Resource Center (UC Davis). The mouse was originally developed as part of the International Mouse Phenotyping Consortium (11). Mice carrying the floxed *Slfn4* gene were then bred to the *Flp* recombinase mouse line to remove the neomycin cassette flanked by FRT sites to leave a *Slfn4* locus floxed at exon 6. To delete the *Slfn4* locus only in *Gli1*-expressing cells, the *Gli1Cre^ERT2^* mouse line was purchased from Jax Labs. The *Gli1Cre^ERT2^* mice were generated by recombination of the *CreERT2* cassette into the 5’UTR of the *Gli1* locus, which allowed expression of endogenous GLI1 protein (12). Genotyping was achieved using the following primers for the *Slfn4^FLEx6FL^* mice:

forward: 5’-AGCATAGGGGACAGATGGGATGG;

reverse: 5’-TTTTATGTGTATGGGTGTTTTGTCTGC

After breeding the *Gli1Cre^ERT2^* mice to the *Slfn4^FLEx6FL^* line, the hybrid mice were injected intraperitoneally with Tamoxifen (TX, 0.2 mg/g body weight) 2 weeks prior to *H. felis* inoculation and then once each month until euthanized. Studies were performed on a mixed group of male and female mice that were cohoused with their littermates that were used as controls. All mice were housed under the same specific pathogen-free conditions.

### Single cell preparation

Eighteen mice infected with *H. felis* for 6-month and their uninfected counterparts were necropsied. PBMCs were isolated from ~1mL of whole blood using a Ficoll gradient according to the manufacturer’s instructions (Ficoll-Paque). The single cell suspension isolated from mouse gastric stomach has been described previously (13). The single cell suspension was flow-sorted to collect viable CD45^+^ and Propidium iodide negative (PI^-^) (Abcam, #ab14083) immune cells, using an Aria III flow sorter (BD) prior to generation of the scRNA-seq library.

### Single-cell RNA sequencing and read processing

Each cell suspension was subjected to 3′ single-cell RNA sequencing using 10X Chromium Controller with accompanying Chromium Next GEM Single Cell 3’ Kit v.3.1 (10X Genomics, Pleasanton, CA), following the manufacturer’s instructions. Libraries were sequenced on a NextSeq Illumina 5000 platform and mapped to the GRCm38/91 mouse reference genome using the 10x Cell Ranger analysis tool.

### Quality control, cell-type clustering and identification

To filter out low-quality cells, we applied quality measures on raw gene-cell-barcode matrix for each cell: mitochondrial genes ≤20%, gene count ranging from 200 to 5,000. Finally, 13,076 cell transcriptomes from PBMCS and 8,394 cell transcriptomes from stomachs were retained for the downstream analysis. Filtered cells were clustered and projected as t-SNE (t-Stochastic Neighbor Embedding) plots using the Loupe Browser (10X Genomics, version 6.2.0), which uses the nonlinear dimensionality reduction method with modifications by 10X Genomics. The major cell types were chosen by initial exploratory inspection of the differentially expressed genes (DEGs) of each cluster. The DEGs were identified by the Loupe Browser. To assign one of the major cell types to each cluster, we scored each cluster by the normalized expression of the canonical markers as shown in **Supplementary table S1**. The highest scored cell type was assigned to each cluster. The clusters assigned to the same cell type were lumped together for the GO enrichment analysis.

### Gene ontology enrichment analysis

GO enrichment analysis of upregulated genes in *Slfn4^+^* subsets were analyzed with ShinyGO V0.76.3 (designed by South Dakota State University) (14). The top 10 or 20 significantly enriched GO (−log10 FDR (Benjamini-Hochberg adjusted p-values) <0.5) terms in molecular functions or biological process were shown. Upregulated genes were identified by 10X Loupe browser (log2 FC>0.58, P ≤ 0.05) relative to all the other subsets.

### Cell culture

Primary myeloid cells prepared from the *Slfn4*-tdTomato (*Slfn4*-tdT) mice were cultured in DMEM media with 10% FBS at 37°C and treated with 100 nM of tamoxifen dissolved in DMSO for 24 h to induce Cre recombinase activity ex vivo (13). Thioglycollate-elicited peritoneal myeloid cells (PCs) were isolated from *Slfn4*-tdT mice as described previously (13). These cells were treated with IFNα (800 U/ml), sildenafil (1-100 µM) or KT5823 (10µM) (Sigma, # 420321) for 24 h; or transfected with *Slfn4* siRNA using Lipofectamine RNAimax reagent (ThermoFisher, #13778150) for 48h prior to further analysis.

### T cell suppression assay

Stomach Slfn4^+^ cells isolated from *Slfn4*-tdT mice were transfected with *Slfn4* siRNA or scrambled siRNA using Lipofectamine RNAiMax reagent for 48 h or treated with sildenafil at different doses for 24 h. Cell pellets were collected for the coculture experiment. T cells from the mouse spleen were isolated using the EasyStep Mouse T Cell Isolation Kit (StemCell Technologies) and pre-stained with carboxyfluorescein diacetate succinimidyl ester (CFSE) using the CellTrace CFSE Cell Proliferation Kit (Molecular Probes, Cat. C34554). T cells were cultured in RPMI 1640 supplemented with 10% FCS, 2 mM L-glutamine, 1 mM sodium pyruvate, 100 mM nonessential amino acids, 5 mM HEPES free acid, 10 ml of 0.05 μM β-mercaptoethanol, and 100 U/mL penicillin/streptomycin with 10% heat-inactivated FBS. To stimulate proliferation, T cells were cultured with anti-CD3/CD28–coated sulfate latex beads in the medium. Suppression of T cell proliferation was assayed after the addition of treated SLFN4^+^ cells as described above for 3 days at a T cell/SLFN4^+^ cell ratio of 10:1. Cell proliferation was analyzed using the FACS Canto II cell analyzer (BD).

### Western blot analysis

Protein extracts from cells were collected in RIPA buffer (Pierce, Rockford, IL). Tissue from the corpus was homogenized in RIPA buffer. Western blots were performed by re-suspending proteins in sample buffer, then resolving on Novex 4–20% Tris-Glycine gels (Invitrogen) using Tris running buffer, and then transferred to PVDF membrane using the iBlot Dry Blotting System (Invitrogen) according to the manufacturer’s instructions. The membranes were blocked in 5% non-fat milk for 1 h at room temperature and then sequentially probed overnight at 4°C with primary antibodies to SLFN4 peptides (custom made by GenScript), NOS2 (Abcam, #ab283655), CHOP (Abcam, #ab11419), cleaved Caspase-3 (Cell Signaling, #9661), β-Tubulin (Cell signaling, #5346S), GAPDH (MA5-15738, ThermoFisher).

### Nucleotide extraction and ATP/GTP assay

The extraction of nucleotides was achieved according to a previously published method (15). The cells were washed three times with cold PBS, collected by centrifugation and the cell pellets deproteinized with an equal volume of 1% trichloroacetic acid on a vortex-mixer for 20s. The acidified cell extracts were centrifuged at 3000 rpm for 10 min at 4°C. The supernatants collected were neutralized to pH 7.5 using 2M Tris, vortexed for 60s for ATP/GTP analysis. The GTPase-Glo™ assay is designed to measure GTPase activity based on the amount of GTP that is present following a GTPase reaction in a two-step assay: converting the remaining GTP into ATP (using GTPase-Glo™ Reagent), and then measuring ATP in a luciferase reaction (using Detection Reagent). The GTPase-Glo™ Reagent from the kit uses the NDPK enzyme to convert GTP to ATP. Addition of GTPase-Glo™ Reagent with the NDPK enzyme to our cell lysates followed by the Detection Reagent detected total GTP+ATP levels in the cell lysates. Parallel wells with the same cell lysate and the GTPase-Glo™ Reagent without NDPK enzyme (GTPase-Glo™ Buffer (V765A) from the kit), followed by the Detection Reagent measured only ATP levels. The level of GTP was calculated as follows (GTP) = (GTP+ATP) – (ATP).

### Assessment of intracellular reactive oxygen species

The intracellular ROS levels were determined using H2DCFDA dye (Abcam, #ab113851). The cells were incubated with 10µM H2DCFDA at 37°C for 45 min followed by two PBS washes. Upon cleavage of the acetate groups by intracellular esterases and oxidation, the nonfluorescent H2DCFDA was converted to the highly fluorescent 2’,7’-dichlorofluorescein (DCF). The fluorescence was analyzed by the BD FACS Canto II cell analyzer.

### Histological analysis

Five-micron paraffin sections were prepared, de-paraffinized in a xylene-alcohol series, followed by rehydration in phosphate-buffered saline (PBS). Antigen retrieval was performed using 10 mM citric acid buffer (pH 6). Peritoneal myeloid cells were cultured on chamber slides (ThermoFisher, # C6932). Slides were washed in 0.01% Triton X-100 in PBS twice, incubated for 1 hour with the serum of the animal in which the secondary antibody was raised. Next, slides were incubated with the following antibodies overnight: lectin GSII from *Griffonia simplicifolia* (Alexa Fluor™ 488-conjugate, ThermoFisher, #L21415), HK/ATP4β (Sigma, #A274), intrinsic factor (LSBio, #LS−C334972), cleaved Caspase-3 (Cell Signaling, #9661), NOS2 (Abcam, #ab283655). Alexa Fluor-conjugated secondary antibodies (Molecular Probes, Eugene, OR; Invitrogen, Carlsbad, CA) were used to detect primary antibodies at a dilution of 1:500. Slides were mounted with Prolong gold anti-fade reagent with DAPI (Life Technologies, Rockville, MD). Immunofluorescence was visualized using the Nikon Eclipse E800 microscope (Nikon, Tokyo, Japan).

For the analysis of mouse mucous cell metaplasia (loss of parietal cells with replacement by mucous-type cells), the presence of “foamy”-appearing epithelial cells in 1 to 2 glands was scored 1, foamy cells in most fields but less than the entire field was scored 2, and the entire glandular epithelium replaced by foamy mucous cells was scored 3. The weighted score = [(number of fields with a score =1) + (2x number of fields with a score =2) + (3x number of fields with a score =3)]/(total number of fields x 3). For each slide, 6 to 10 high power fields at a magnification of 200X were scored.

### Annexin V staining

Annexin V staining was used for detecting apoptotic cells. Live cells were treated with Annexin V, Alexa Fluor 488 conjugate (Invitrogen, #A13201) for 15 min to identify apoptotic cells by flow cytometry.

### cGMP assay

Cells treated with or without Sildenafil were homogenized in 0.1M HCl. Homogenates were incubated for 10 min and an aliquot was saved for protein determination. Homogenates were centrifuged at 600 x g for 10 min and then the supernatant was acetylated using 0.01 mL of freshly made acetylation reagent (0.5mL of acetic anhydride mixed with 1mL of Triethylamine) and 0.2 mL of supernatant. The cGMP levels present in the supernatant was determined using an ELISA kit (ThermoFisher, #EMSCGMPL). An aliquot was assayed from 0.1mL of acetylated sample run in duplicate using 0.08 to 50 pmol/mL of acetylated cGMP standards according to the manufacturer. After 2 h of incubation at room temperature, samples were washed, 0.2mL of p-NPP substrate solution was added to each well and incubated for additional 1h at room temperature. The reaction was stopped by adding 0.05 mL of stop solution to each well and the optical density (OD) was read at 405 nm. cGMP concentration was calculated by analyzing the data using a 4-parameter logistic curve-fitting program. The final concentration of cGMP was normalized to protein and expressed as fmol/mg protein.

### Mouse TNF alpha and IL-1 alpha

Protein was extracted by homogenizing gastric tissue in RIPA buffer supplemented with proteinase inhibitor (ThermoFisher, #78425) and the supernatant was used in the assay. TNFα levels were measured using mouse ELISA kit (ab113344) and IL1α levels were measured using mouse ELISA Kit (R&D MLA00), per the manufacturers' instructions.

### Statistical analysis

For qPCR and ELISA, statistical analysis for significance was performed using Kruskal-Wallis one-way ANOVA with Dunn’s test for multiple comparisons. All data were expressed as the median with the interquartile range. P<0.05 were considered significant. The number of samples per group and replicate experiments are indicated in the figure legends.

### Study approval

All animal care and experimental procedures were conducted in compliance with the guiding principles in the Care and Use of Animals and the Animal Management rules of the University of Arizona Institutional Animal Care and Use Committees.

## Results

### scRNA-seq profiling maps Slfn4 expression to type 1 IFN-regulated MDSCs in Helicobacter-infected stomachs

**Figure 1:**
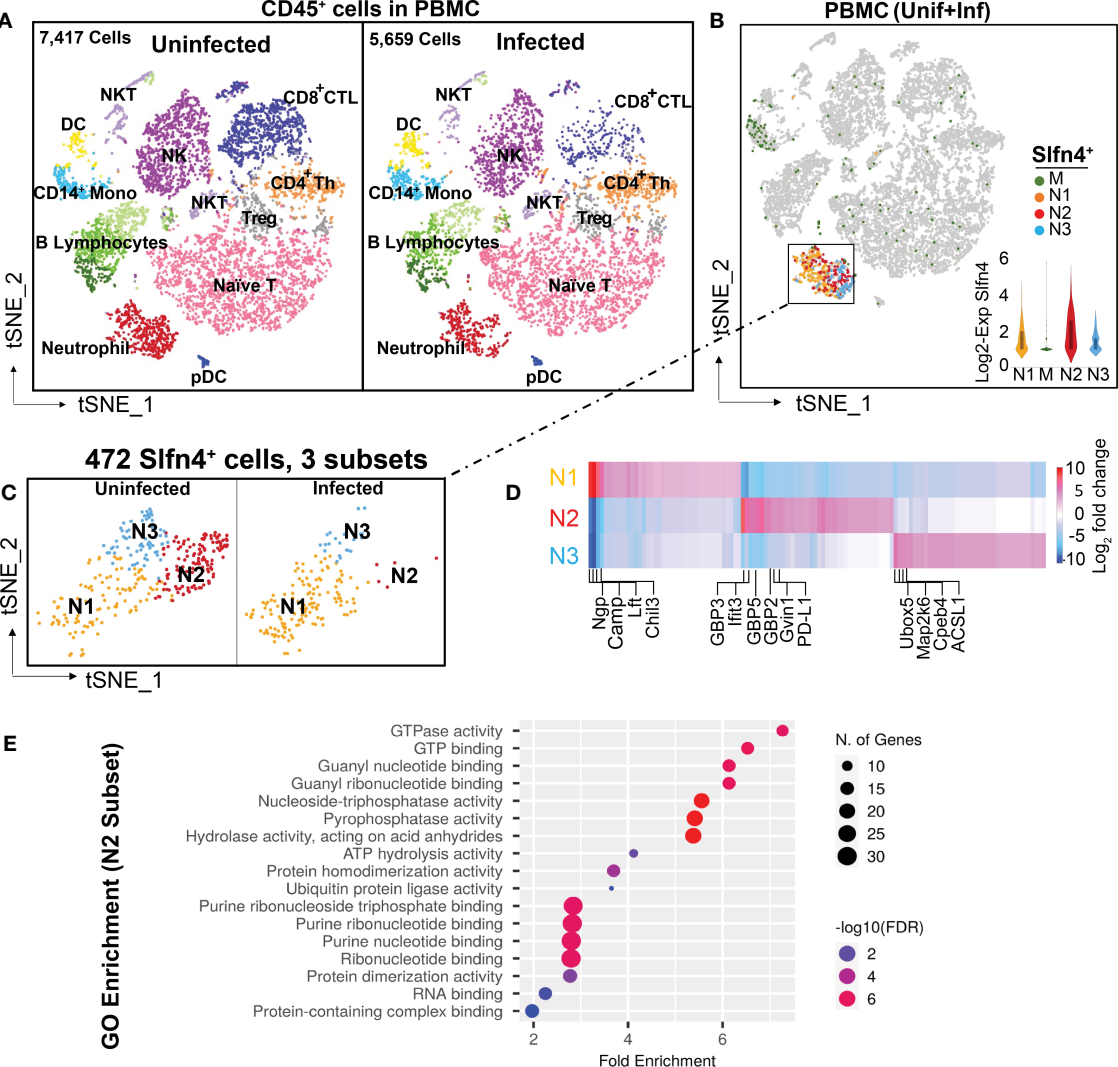
Single cell transcriptional profiling of FACS-sorted CD45^+^ immune cells from mouse peripheral blood mononuclear cells (PBMCs). **(A)** t-SNE visualization of FACS-sorted CD45^+^ PBMC immune cells from uninfected (7,417 cells) and *H*. *felis*-infected mice (5,659 cells). Major cell lineages are defined by markers as shown in **Supplementary table S1** and color coded as indicated. **(B)** Single cell *Slfn4* gene expression in PBMC immune cells (uninfected + infected) were color coded for 4 subsets M (green), N1 (Maize), N2 (red) and N3 (blue). Violin plots depict *Slfn4* expression levels (log2 transformed) in each subset. **(C)** *Slfn4* ^+^ cells (472 cells) of N1-N3 subsets from boxed area in **(B)** were separately shown for uninfected and *H*. *felis*-infected PBMCs. **(D)** Heatmap of differentially expressed genes between N1-N3 subsets. Representative enriched genes in each subset are indicated. The color scale is log2 FC from -10 (blue) to 10 (red). Differentially expressed genes (DEGs) were identified by the 10X Loupe browser (FDR ≤ 0.05) relative to all the other subsets. **(E)** Gene ontology (GO) enrichment analysis of upregulated genes in the N2 subset of *Slfn4* ^+^ cells. The top 20 significantly enriched GO molecular functions (−log10 (p-value) are shown. Color scale: -log10 FDR (Benjamini-Hochberg adjusted p-values); the size of circles: gene numbers; X-axis, fold enrichment; Y-axis, GO term (p < 0.05). Upregulated genes were identified by 10x Loupe browser with (log2 FC>0.58, FDR ≤ 0.05) relative to all the other subsets.

**Figure 2:**
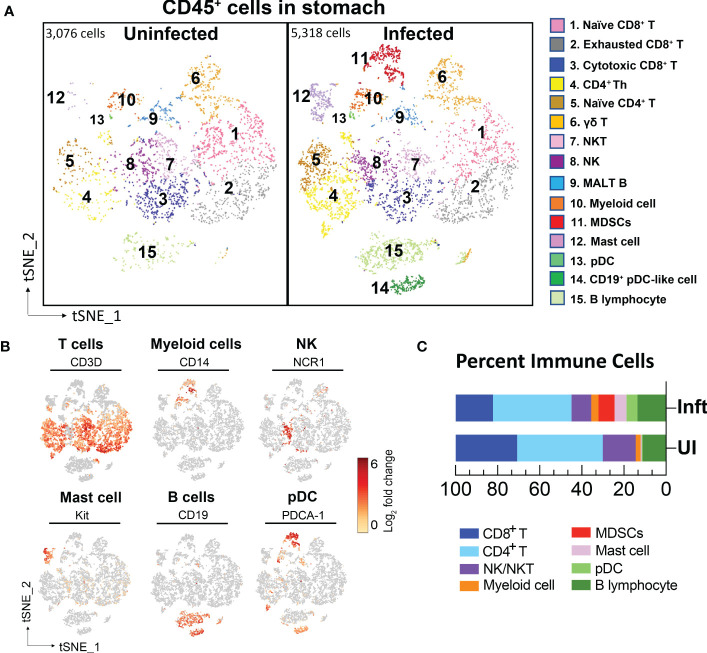
Single cell transcriptional profiling of FACS-sorted CD45^+^ immune cells from mouse stomachs. **(A)** t-SNE visualization of immune cells from uninfected- (3,076 cells) and *H*. *felis*-infected mouse stomachs (5,318 cells). Cell lineages were defined by canonical markers as shown in **(B)** and **Supplementary table S1**. **(C)** Percent of major immune cell lineages in the uninfected and infected stomachs were shown.

To define the identity of Slfn4^+^ cell populations in *H. felis*-induced gastric metaplasia, single cell RNA sequencing (scRNA-seq) was performed on FACS-sorted CD45^+^ immune cells from the PBMCs and stomachs from *H. felis*-infected and uninfected mice. After sorting, cells were washed and rapidly processed using the 10x Genomics Chromium platform. After filtering scRNA-seq data to exclude putative cell doublets and cells with high mitochondrial signatures, a total of 13,076 cell transcriptomes from peripheral blood mononuclear cells (PBMCs) and 8,394 cell transcriptomes from stomachs were analyzed from uninfected or *H. felis*-infected mice. These data were visualized by tSNE projections and partitioned into several major cell clusters (**Figures 1**, **2**).

PBMCs from *H. felis*-infected mice expressed higher levels of IgG-related genes (**Supplementary figure S1**), but otherwise showed no significant differences among the major immune cell populations (**Figure 1A**). Slfn4^+^ cells isolated from PBMCs were mainly concentrated in the neutrophil cluster, and at lower levels in the monocyte cluster (**Figures 1B, C**). Sub-clustering of Slfn4^+^ cells revealed 4 subsets: three neutrophil clusters N1-N3 and the monocyte cluster M exhibiting much lower *Slfn4* expression (**Figures 1B, C**). Interestingly, the N2 subset was identified in the uninfected PBMCs, but was nearly absent in the PBMC from infected mice. This N2 subset exhibited strong transcriptional signatures for type I interferon regulated responses such as GTPases, including GBPs, IFITs and GVIN1 (**Figure 1D**; **Supplementary table S1**). GO enrichment analysis also strongly suggested that the cellular function of the N2 subset correlated with the GTPase/GTP pathways (**Figure 1E**).

**Figure 3:**
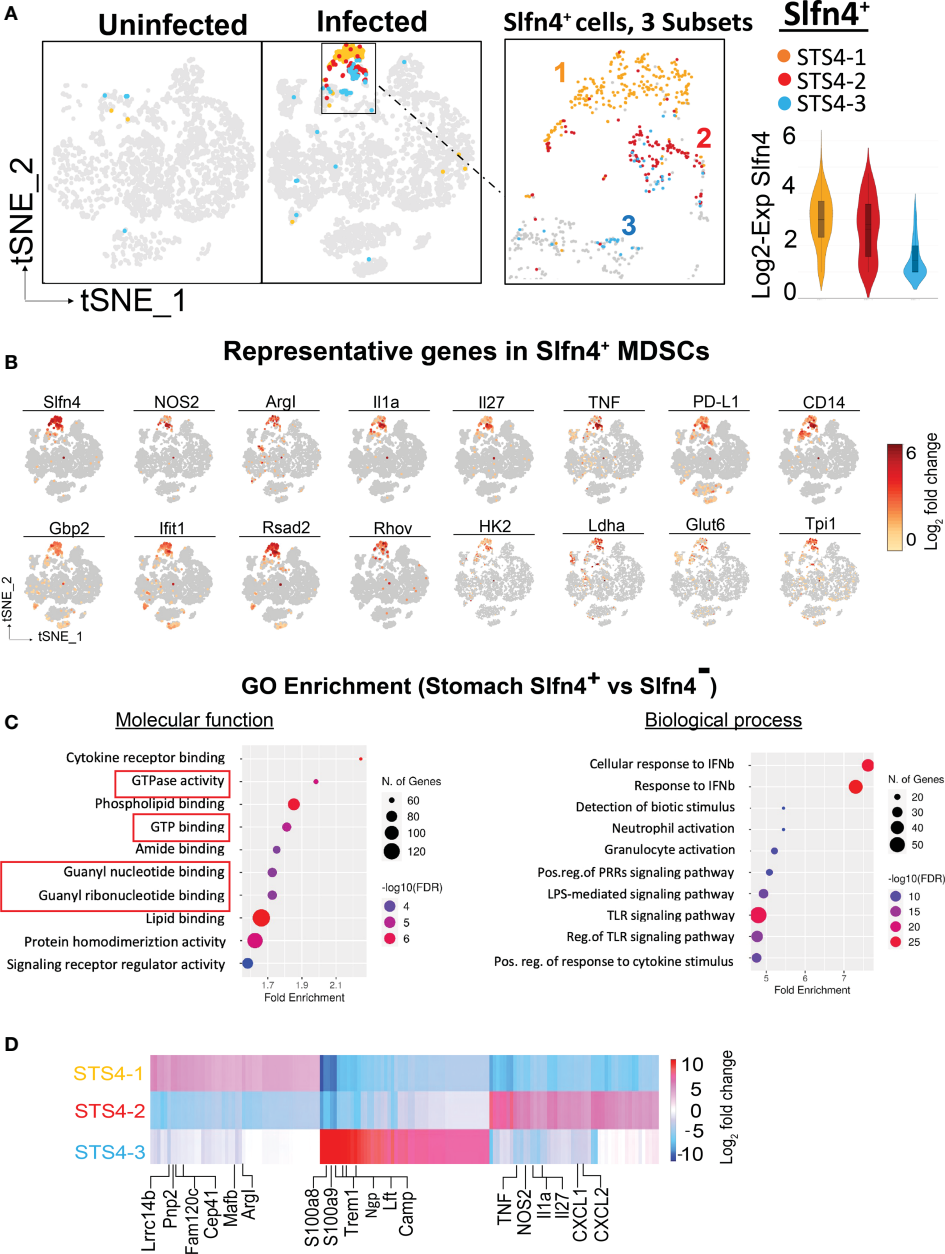
Single cell transcriptional profiling of Slfn4+ immune cells from mouse stomachs. **(A)** Stomach single cell *Slfn4* gene expression was color coded for 3 subsets STS4-1(Maize), STS4-2(red) and STS4-3 (blue). The boxed area with *Slfn4* gene expression was enlarged on the right. Violin plots depict *Slfn4* expression levels (log2 transformation) in each subset. **(B)** Representative up-regulated genes color-coded fold-change expression levels (gray to red of 12 representative genes in Slfn4^+^ -MDSCs). **(C)** GO enrichment analysis of upregulated genes in stomach *Slfn4* ^+^ vs *Slfn4* ^-^ immune cells. The top 10 significantly enriched GO (−log10 (p-value)) terms of molecular functions and biological process were shown. Upregulated genes in Slfn4^+^ were identified by 10x Loupe browser with (log2 FC>0.58, FDR ≤ 0.05) relative to Slfn4^-^ cells. **(D)** Heatmap of DEGs between 3 subsets of STS4. Representative enriched genes in each subset were indicated. The color scale is from log2 FC-10 (blue) to log2 FC 10 (red). DEGs were identified by 10X Loupe browser (FDR ≤ 0.05) relative to all the other subsets.

The major known immune cell lineages in the stomach were identified for both uninfected and infected mice (e.g., T/B lymphocytes, NK cells, myeloid cells) (**Figures 2A–C**; **Supplementary table S1**). However, myeloid derived suppressor cells (MDSCs) expressing *Nos2*, *ArgI*, *TNF*α and *IL-1*α were only present in the infected stomachs (**Figures 2A**, **3B**). Mast cells, which are well known to be critical for MDSC immune suppression (16, 17), also increased in the infected stomachs. We previously reported that plasmacytoid dendritic cells (pDCs) are a major cellular source of type-I interferons and is the major inducible activator of *Slfn4* gene expression (18). There was a slight increase in the classic pDC population (*Pdca-1^+^Irf7^+^Ly6c2^+^siglec-H* ^+^), whereas a distinct subset of pDCs (*Pdca-1^+^Irf7^+^Ly6c2* ^+^) co-expressing the B cell lineage marker CD19 emerged in large numbers in the infected stomachs (**Figure 2**). This distinct subset has been reported to be associated with T cell suppression (19). As expected, *Slfn4* ^+^ cells were significantly higher in the infected stomachs and mainly concentrated in the MDSC cluster, in contrast to a few scattered cells in the myeloid cell population STS4-3 (**Figure 3A**). As shown in **Table 1** and **Figure 3B**, stomach *Slfn4^+^* immune cells highly express MDSC-related enzymes *Nos2* and Arg-I, and MDSC-related cytokines such as *Il-27 (* 20), *IL-1*α *(* 21), *Tnf (* 22), *Ccl7 (* 23), and *Pd-*L1, which is usually highly elevated in activated MDSCs and induces T cell exhaustion by binding to the ligand PD-1 (24, 25). Remarkably, IFNα-inducible GTPases including *Iigp1, Gvin*, and guanylate binding proteins (GBPs), which are all large dynamin-type GTPases that hydrolyze GTP to GDP were also highly induced in *Slfn4^+^* cells (**Figure 3B**), consistent with a higher state of oxidative phosphorylation in MDSCs (26). An enrichment of enzymes PDE6g, PDE7b and PDE8b revealed potential susceptibility of these cells to PDE inhibitors. Some other genes involved in energy metabolism, like Hexokinase (*Hk*), Triose phosphate isomerase (*Tpi*), Lactate dehydrogenase (*Ldh*) and glucose transporter (*Glut*), are also highly expressed in the MDSCs (**Figure 3B**),which can provide sufficient energy for MDSCs’ suppressive function (27).

**Table 1:** Representative up-regulated genes and log2-transformed fold change in Slfn4^+^ cells relative to Slfn4^-^ cells isolated from stomach.

Gene	Log2 Fold	Gene	Log2 Fold
Il27	6.325445271	Gbp2b	6.177244225
Nos2	6.282060633	Rsad2	5.49936616
Ccl7	6.25349148	Rhov	5.16763074
Il1a	5.59985427	Gbp5	4.6348831
Cxcl10	5.481444081	Rab13	4.33400084
Tnf	5.129615283	Arl11	4.1734745
Ccl9	4.503350498	Gbp3	4.10883214
Csf3r	4.81509184	Ifit1	4.06939232
Cxcl2	4.36876968	Iigp1	4.03443034
Cd40	3.70169932	Ifit2	3.99180178
Arg1	3.69072616	Gbp2	3.60997957
Ccl2	3.59689449	Ifit3	3.3023143
PD-L1	2.79869652	Pde6g	2.38724287

The most enriched GO terms in the up-regulated genes of *Slfn4^+^* cells were highly involved in GTPase and GTP binding, response to type-I interferons, toll-like receptor signaling pathway and granulocyte activation (**Figure 3C**), consistent with the known induction of *Slfn4* in the MDSCs by type-I interferons (13, 18, 28). Coincidently, these *Slfn4^+^* cells in the infected stomachs shared transcript like those in the N2 subset that disappeared in the infected PBMC immune cells (**Figures 1D, E**), except that stomach *Slfn4^+^* cells highly expressed MDSC markers. These observations suggested that the N2 subset of *Slfn4^+^* cells may migrate from the peripheral blood to the stomachs upon *H. felis* infection and then acquired the MDSC phenotype.

Further sub-clustering of stomach *Slfn4^+^* cells revealed 3 subsets (**Figure 3A**). By comparing gene signatures between each subset (**Figure 3D**; **Supplementary table S1**), the STS4-3 subset with lower *Slfn4* expression was enriched in classic neutrophil transcripts (*Ngp, Camp, Ltf, S100a8* and *S100a9*) that were common to both the uninfected and infected stomachs, representing the basal level of *Slfn4* in the stomach. STS4-1 and STS4-2 subsets were highly enriched in transcripts for *Slfn4* and type-I IFN inducible GTPases (**Figure 3D**). Specifically, STS4-1 was enriched in *Arg-I* and monocyte markers (e.g., *Lrrc14b, Pnp2, Cep41, Mafb*), suggestive of M-MDSCs. By contrast, the STS4-2 subset with highly expressed granulocyte markers (*Cxcl1, Cxcl2*) and MDSC cytokines *Tnf*, *IL1a* as well as *Nos2*, suggestive of G-MDSCs (**Figure 3D**). We inferred that these two subsets migrated from the circulation to the stomach upon *Helicobacte*r infection where they polarized into MDSCs.

### Slfn4 knockdown or sildenafil reduced Slfn4^+^-MDSC immunosuppression by depressing GTP levels

**Figure 4:**
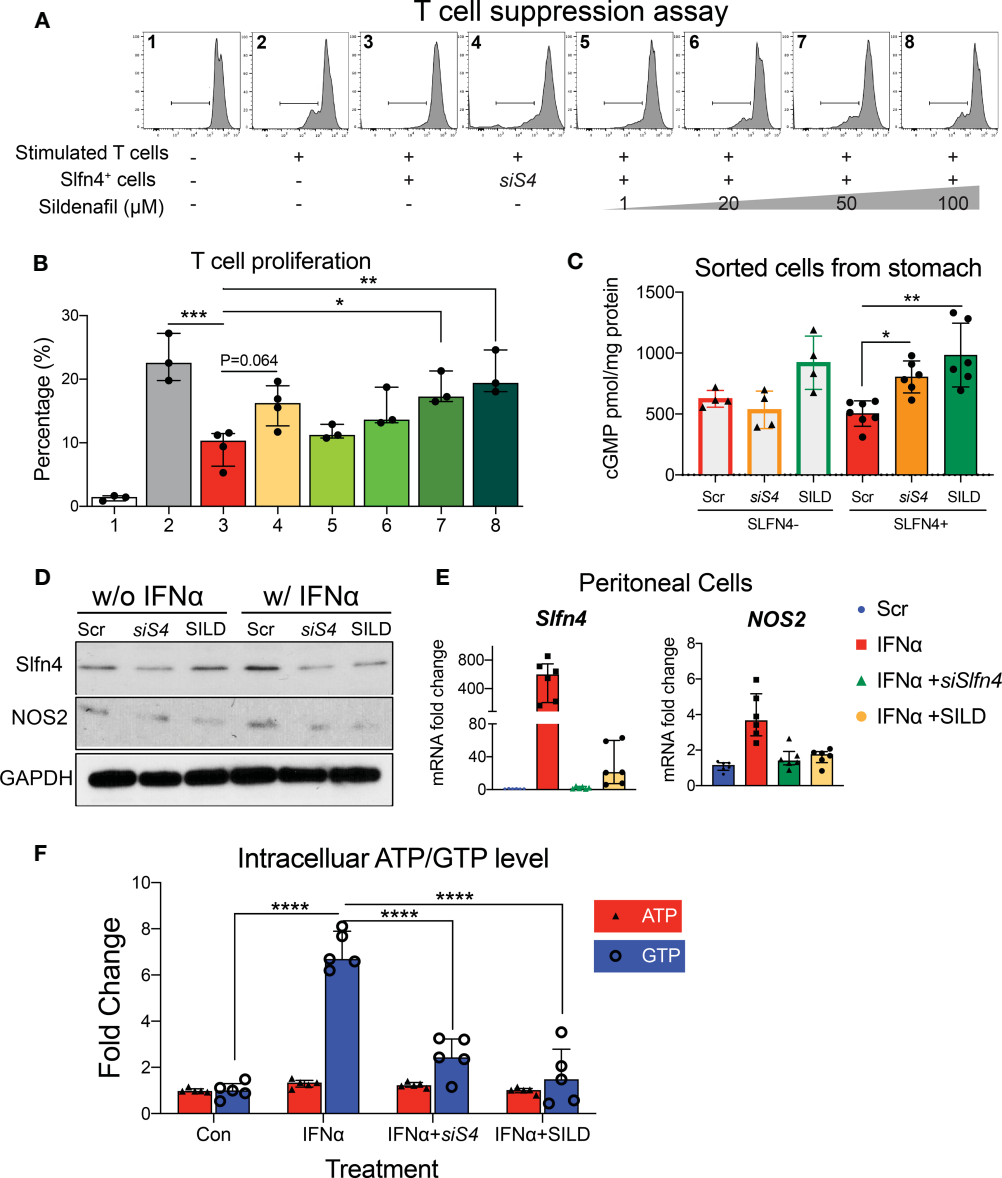
Slfn4 suppression or Phosphodiesterase 5/6 inhibitor Sildenafil treatment interrupted GTP/GTPase/cGMP pathway and reduced Slfn4+ MDSCs’ immunosuppressive function. Stomach Slfn4-tdTomato^+^ cells were flow-sorted from *H*. *felis*-infected Slfn4-tdTomato mice and then transfected with Slfn4 siRNA (scrambled siRNA as control) or treated with different doses of Sildenafil as indicated for 1 day before coculture with splenic T cells. **(A)** Flow cytometry was performed to analyze CFSE-based T cell proliferation and quantified in the bar graphs. **(B)** Kruskal-Wallis one-way ANOVA with Dunn’s test for multiple comparisons. N=3-4 experiments. **(C)** Cellular cGMP levels were detected by ELISA. N=4-7 experiments. Thioglycollate-elicited peritoneal myeloid cells were treated with IFNα (800U/ml), combined with Sildenafil (50 µM) or *Slfn4 siRNA* transfection. Expression of SLFN4 and NOS2 were determined by **(D)** Western blot (representative images); **(E)** qPCR, **P < 0.01 over IFNα treatment alone. Mag: 200X. GAPDH as a loading control. **(F)** Intracellular ATP and GTP levels after different treatments determined by GTPase-GLO ELISA. Kruskal-Wallis one-way ANOVA with Dunn’s test for multiple comparisons. N=6 experiments. Horizontal lines represent the median and interquartile range. *P < 0.05, **P < 0.01, ***P < 0.001, ****P < 0.0001.

Since the NO/GTPase pathway components were elevated in the *Slfn4^+^*-MDSCs, we tested if blocking this pathway genetically or pharmacologically might mitigate the suppressive effects of *Slfn4^+^*-MDSCs. Considering that SLFN4 contains a GTPase-like domain at its C-terminus (10) and might contribute to GTPase activity, we knocked down *Slfn4* using siRNA in cultured *Slfn4*-tdT^+^ flow-sorted from *H. felis*-infected stomachs which reduced their suppressive effect on CTL proliferation (**Figures 4A, B**), indicating that *Slfn4* is essential to the MDSC function. Since cGMP is a key player in the NO/GTPase pathway and activated by increased production of NO (29), we performed ELISA to detect intracellular concentration of cGMP levels in these sorted cells. As shown in **Figure 4C**, cGMP level in stomach Slfn4^+^ cells were not higher compared to Slfn4^-^ cells as expected, however, cGMP went up higher after Slfn4 was knocked down by *siRNA*, suggesting Slfn4 plays a role in cGMP regulation. Therefore, we assessed whether the well-known type 5/6 PDE inhibitor sildenafil (SILD) blocked cGMP hydrolysis and therefore interrupted Slfn4^+^ MDSC function. Cultured *Slfn4*-tdT^+^ cells isolated from the *H. felis*-infected stomach were treated with SILD for one day, which increased their intracellular levels of cGMP (**Figure 4C**). Furthermore, dose-dependently SILD reversed the inhibition of CTL proliferation mediated by *Slfn4^+^* cells (**Figures 4A, B**). Thus, both SILD and the knockdown of *Slfn4* effectively inhibited *Slfn4^+^*-MDSCs function.

**Figure 5:**
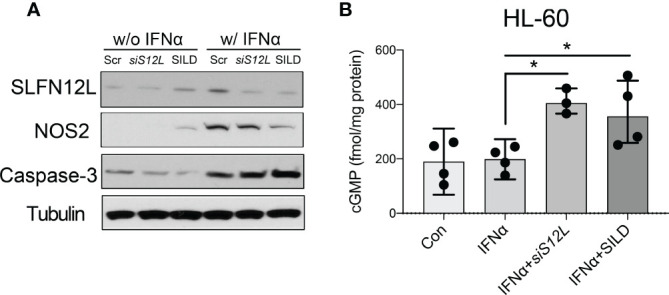
The effect of Sildenafil and Slfn12L knockdown on human HL-60 cells. HL-60 cells were treated with IFNα (800 U/ml), sildenafil (50 µM) or transfected with *SLFN12L siRNA*. **(A)** Representative western blot of SLFN12L, NOS2 and Caspase-3. GAPDH as a loading control. **(B)** Intracellular cGMP level detected by ELISA. Kruskal-Wallis one-way ANOVA with Dunn’s test for multiple comparisons. N=3 experiments. Horizontal lines represent the median and interquartile range. *P < 0.05.

Since NOS2 is an enzyme essential for MDSC function, generation of NO and sGC activation, we further examined the effect of SILD and reduced *Slfn4* on *Nos2* expression. We have shown previously that IFNα is the most potent inducer of *Slfn4* gene expression and is sufficient to polarize thioglycolate-elicited peritoneal myeloid cells (PCs) to acquire MDSC suppressor function *in vitro (* 13). Both *Slfn4* knockdown and sildenafil blocked IFNα-mediated induction of SLFN4 and NOS2 (**Figures 4D, E**), consistent with the suppression of MDSC function (**Figures 4A, B**). GTP versus ATP levels were quantified and revealed that IFNα increases GTP but not ATP levels (**Figure 4F**). Both SILD and *Slfn4* knockdown suppressed intracellular GTP levels, coincident with the loss of NOS2 and MDSC function, suggesting that loss of guanine nucleotides in these *Slfn4^+^*-MDSCs was sufficient to block NOS2 function. In addition, SILD suppressed IFNα-mediated induction of ortholog SLFN12L and NOS2 in HL-60 cells and increased intracellular cGMP level, suggesting that the two orthologs exhibit similar function (**Figures 5A, B**).

**Figure 6:**
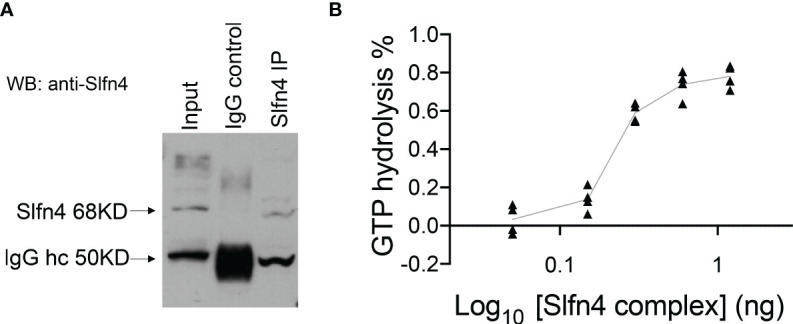
Precipitated SLFN4 complex exhibits GTPase activity. **(A)** Immunoprecipitation of SLFN4. Extracts from RAW 264.7 cells treated with IFNα (800U/ml) for 16h were immunoprecipitated using an anti-SLFN4 antibody followed by immunoblotting using anti-SLFN4 to confirm the presence of SLFN4 in the cellular lysate. **(B)** GTPase-Glo assay (Promega) shows that the precipitated SLFN4 complex exhibits GTP hydrolyzing activity in a dose dependent manner. N=4 experiments.

Since SLFN4 contains a GTPase domain at its C-terminus, we examined whether immunoprecipitated SLFN4 from IFNα-treated RAW264.7 immune cells exhibited GTPase activity (**Figure 6A**). The SLFN4-containing complexes immobilized on beads hydrolyzed GTP in a dose-dependent manner (**Figure 6B**), suggesting that SLFN4 protein may possess GTPase activity either alone or in a complex with other GTPases.

### Slfn4 knockdown or sildenafil treatment enhanced IFNα-induced ROS generation and apoptosis *via* PKG signaling

**Figure 7:**
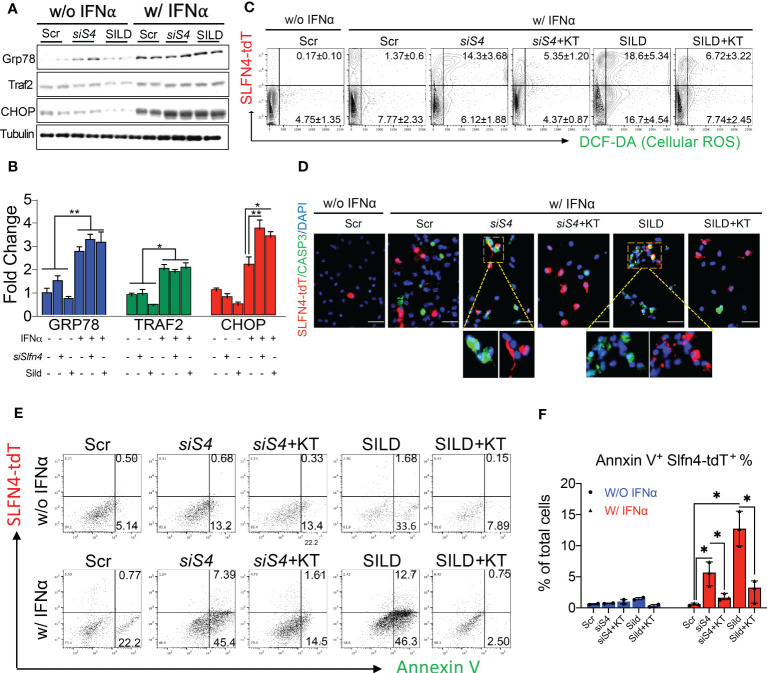
Slfn4 suppression or Sildenafil augment ROS and apoptosis of myeloid cells under stress induced by IFNα *via* PKG signaling pathway. Peritoneal myeloid cells were collected from Slfn4-tdTomato mice and treated with Tamoxifen *in vitro* to induce tdTomato fluorescent protein, combined with other treatments as indicated. **(A)** ER stress markers GRP78, TRAF2 and CHOP were detected by Western blot. Tubulin was used as the loading control. **(B)** Bar graphs show quantitation using ImageJ. **(C)** Cellular ROS determined by DCF-DA staining (green). Cell apoptosis was determined by **(D)** IF staining of Caspase-3 staining (green), Mag: 400x; **(E)** Annexin V flow cytometry analysis. **(F)** Bar graphs show quantitation of percent of AnnexinV^+^Slfn4-tdT^+^ cells determined by flow. KT, PKG inhibitor KT5823 (10uM). Scr, scrambled control. N=3 expts. Kruskal-Wallis one-way ANOVA with Dunn’s test for multiple comparisons. Horizontal lines represent the median and interquartile range. *P < 0.05, **P < 0.01.

MDSCs generate reactive oxygen species (ROS) as part of their T cell suppressor function (30, 31). Accumulation of cGMP activates protein kinase G (PKG) and subsequently apoptosis *via* mechanisms that involve ER stress and excess ROS generation (32, 33). Using a primary culture of PCs, we found that IFNα alone induced the ER stress markers GRP78, TRAF2 and CHOP (**Figures 7A, B**). Moreover, the increase in the CHOP transcription factor was significantly enhanced with *Slfn4* knockdown or SILD treatment. Using PCs prepared from *Slfn4*-tdT^+^ mice, we found that IFNα treatment induced ROS and apoptosis primarily in the PCs that did not express SLFN4 (**Figures 7C, D**). By contrast, IFNα treatment with *Slfn4* knockdown or SILD enhanced ROS generation and apoptosis ~10 fold over IFNα treatment alone (**Figures 7C–F**). Since cGMP is a cofactor for the cGMP-dependent protein kinase (PKG), we treated the PCs with PKG inhibitor KT5823 and significantly reduced cellular ROS generation and apoptosis initiated by IFNα (**Figures 7C–E**). The SLFN4^+^ cells were more susceptible to cell death if either SLFN4 was knocked down or the culture was treated with SILD (**Figure 7F**). KT-5823 mitigated ROS generation and apoptosis, which implicated a central role for PKG in the demise of these *Slfn4* ^+^-MDSCs.

### Conditional deletion of Slfn4 in Gli1^+^ cells or sildenafil treatment in mice mitigated Helicobacter-induced gastric metaplasia

**Figure 8:**
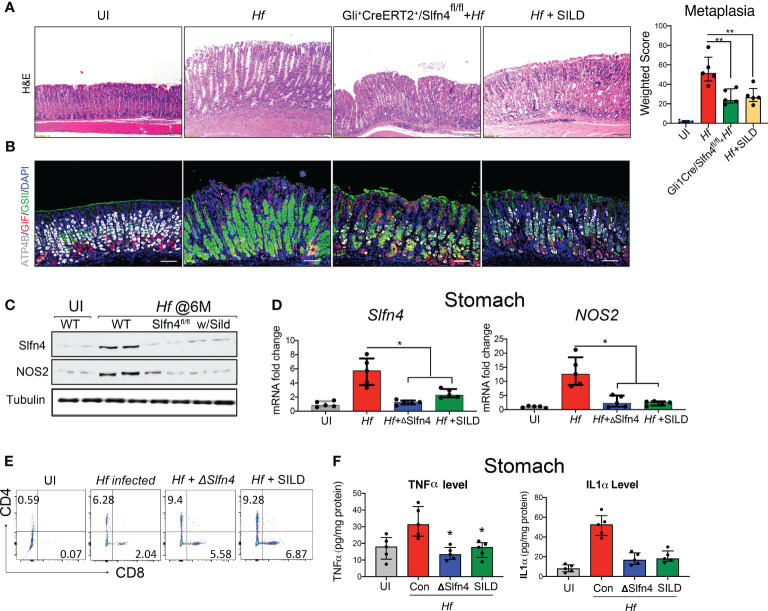
Genetic deletion of Slfn4 in Gli1^+^ cells or Sildenafil treatment mitigated *Helicobacter*-induced metaplasia and reversed T cell suppression. Representative images of gastric mucosa from uninfected WT mice (UI), 4-month-infected WT mice (*Hf*), *Gli^+^CreERT2^+^/Slfn4^fl/fl^* mice and mice with sildenafil gavage by **(A)** H&E staining and **(B)** IF staining for ATP4b (white), GSII (green), gastric intrinsic factor (red). Scale bar, 100 μm. Corpus metaplasia weighted score for N= 5 mice per group. One-way analysis of variance (ANOVA) followed by Friedman test was performed. **(C)** Representative western blot images of SLFN4 and NOS2 proteins with TUBULIN used as a loading control. qPCR of **(D)** stomach *Slfn4* and *NOS2*. **(E)** flow analysis of stomach immune cells on T cell marker CD4 and CD8. **(F)** TNFα and IL1α cytokine levels in the stomachs were measured by ELISA. Kruskal-Wallis one-way ANOVA with Dunn’s test for multiple comparisons. Horizontal lines represent the median and interquartile range for n = 5 mice per group. *P <0.05 compared to *Hf* infected group. **P<0.01.

We previously showed that *H. felis* infected *Gli1^-/-^* mice do not develop spasmolytic protein expressing metaplasia (SPEM) (13). Subsequently, we showed that *Gli1* is only expressed in gastric myofibroblasts and infiltrating myeloid populations during a *Helicobacter* infection and that *Slfn4* is a direct target of GLI1 (34). Therefore, to further define the contribution of *Slfn4^+^*-MDSCs in *Helicobacter*-induced SPEM, the *Slfn4* locus was conditionally disrupted in *Gli1Cre^ERT2^* x *Slfn4^FLEx6FL^* (*ΔSlfn4*) mice and was compared to the gastric histology observed after SILD administration 4 months after inoculating with *H. felis*. After 2 months of SILD treatment, the mice were euthanized at 6 months post-infection. Less SPEM developed in the *ΔSlfn4* mice or after 2 months of SILD treatment as shown by reduced GSII-lectin staining and recovery of some of the H^+^, K^+^-ATPase^+^ parietal cells (**Figures 8A, B**). *H. felis*-induced *Slfn4* and *Nos2* that were suppressed in the *ΔSlfn4* or SILD-treated stomachs (**Figures 8C, D**). Both *ΔSlfn4* and SILD treatment were sufficient to increase the infiltrating gastric T cell population (**Figure 8E**), especially the recovery of CD8^+^ cytotoxic T cell numbers, consistent with impaired *Slfn4^+^*- MDSC function. Since *Slfn4^+^*-G-MDSCs highly express the *Tnf* and *Il1a* cytokines, which contribute to MDSC immunosuppressive activity by inducing *Nos2* (22), we also measured gastric TNFα and IL-1α by ELISA and found that their levels induced by the *H. felis* infection were suppressed by *ΔSlfn4* or SILD treatment (**Figure 8F**).

## Discussion

In the current study, we performed scRNA-seq to identify which immune cells from mouse PBMCs and the *Helicobacter* infected stomachs express *Slfn4*. We observed that SLFN4 is highly expressed in the infected stomach by a mixture of M-MDSCs and G-MDSCs. G-MDSCs displayed higher levels of MDSC-related enzymes ArgI and NOS2, and cytokines, e.g., *Il-27 (* 20), *IL-1*α (21), *Tnf* (22), and therefore may exhibit greater T cell suppression. Both Slfn4^+^ subgroups 1 and 2 strongly express type I interferon response genes, e.g., *Ifit1/3, Iigp1, Gvin*, and *GBPs*. Zilionis et al. previously performed scRNA-Seq using immune cells from human patients as well as mouse models of lung cancer and identified a rare but distinct tumor-associated neutrophil (TANs) subgroup (N2) that shares nearly all the genes identified in our *Slfn4* ^+^ G-MDSCs, including expression of Slfn4 (35). Therefore we propose that the N2 and Slfn4+ G-MDSC cell populations are equivalent. Although their role in chronic infections may be underappreciated, neutrophils are present in the circulation and tumor microenvironment (36, 37). They can polarize into an anti-tumor phenotype with TGFβ or polarize into a pro-tumor phenotype (G-MDSCs) in response to type I IFN (38). The subset of neutrophils with low *Slfn4* expression (STS4-3) may originate from peripheral blood, and transition into “G-MDSCs” (putative pro-tumorigenic N2 neutrophils) which might explain why a subset of *Slfn4^+^* neutrophils with type I IFN inducible genes were nearly absent in the PBMCs of infected mice.

MDSCs usually arise in oxidative-stress prone environments such as in tumors or during infection (26). Elevated levels and continuous production of ROS during oxidative phosphorylation in MDSCs react with NO to form peroxynitrite, which is a potent oxidant that induces apoptosis and suppresses T cell responses but also exhibits toxic effects on MDSCs themselves. MDSCs therefore have lower viability and a shorter half-life in tumor-bearing mice compared to neutrophils and monocytes and rely on the continuous expansion of MDSCs in the bone marrow (39). Here we found that SLFN4 maintained the activity of the NO-GTP-cGMP-GTPase pathway in these MDSCs and precluded these cells from succumbing to the massive ROS generation initiated by IFNα when they acquire MDSC function.

**Figure 9:**
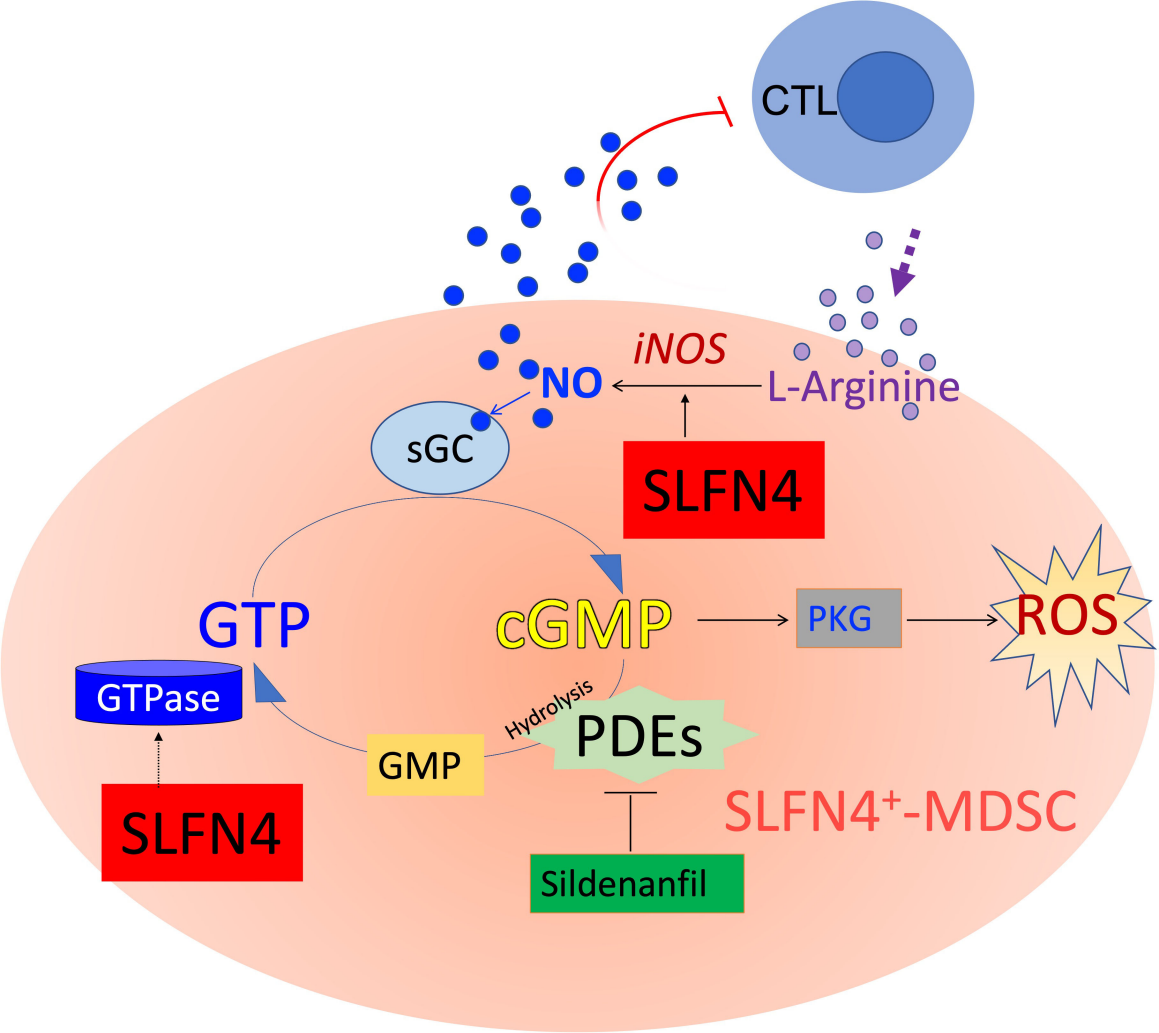
Schematic of cyclic GMP (cGMP) signaling pathway. MDSCs suppress cytotoxic T lymphocytes (CTL) by expressing nitric oxide synthase 2 (NOS2), which metabolizes L-arginine to produce nitric oxide (NO), exerting dual effects on T cells by both depleting the essential amino acid required for T cell proliferation, while also directly influencing T cell signaling and activation. NO is co-factor for soluble guanylate cyclase (sGC), which converts GTP to cyclic guanylate monophosphate (cGMP). cGMP binds to and activates cGMP-dependent protein kinase G (PKG), which in some cells generates ROS and eventually apoptosis. Accumulation of cGMP is rapidly hydrolyzed by phosphodiesterases (such as PDE5/6, a sildenafil target). GTPases hydrolyze GTP to GDP and subsequently to GMP. SLFN4 may contribute to intracellular GTPase activity required to regenerate substrate for the NO-GTP-cGMP pathway and support NOS2 (iNOS, inducible NOS) activity. Maintaining high GTP levels and low cGMP levels shunt substrate away from PKG signaling, ROS generation and ultimately apoptosis.

Nitric oxide produced by NOS2 up-regulates the GTPase pathway (40), whereas GTPase activity is abolished with the accumulation of cGMP (41) (**Figure 9**). A GTPase domain was identified in the tertiary structure of Slfn4/SLFN12L (10) and may function to maintain intracellular GTPase activity, which fuels the NO-GTP-cGMP pathway. However, whether SLFN4 has GTPase activity itself remains to be further elucidated. Interrupting this GTP-cGMP loop either by knocking down SLFN4 or by cGMP-dependent PDE inhibition decreased NOS2, intracellular GTP levels and activated cGMP-dependent kinase (PKG). Intracellular GTP plays a pivotal and distinct role in the regulation of a wide variety of processes including cellular proliferation and maturation (42). In addition, GTP plays an important role in cell fate, differentiation, and apoptosis (43). In the human leukemia cell line K562, the attenuation of GTP by 30-40% induced cell differentiation and augmented intracellular ROS by 60%, which subsequently led to cell death (43). The elimination, differentiation, or functional inhibition of MDSCs in tumor-bearing hosts can restore CD8^+^ T cell responsiveness (44). Accordingly, in our *ΔSlfn4* mice, the loss of this MDSC subset increased the CD8^+^ T cell population in the stomach and mitigated *H. felis*-induced SPEM. Therefore, the role of SLFN4 in regulating GTP/cGMP levels might be essential to sustaining MDSC function.

In this study we also demonstrated that sildenafil, a type-5/6 phosphodiesterase inhibitor, counteracts the suppressive effects of *Slfn4^+^*-MDSCs. PDE5i has been repurposed and tested in human clinical trials for the treatment of malignancies as candidate agents for restoring host immunity *via* MDSC inhibition (45, 46). SILD caused cGMP accumulation and disrupted the NO-GTP-cGMP pathway, which lead Slfn4^+^ MDSCs to undergo apoptosis in a PKG dependent manner. This mechanism is like that proposed for *ΔSlfn4*, since both approaches disrupt the GTP-cGMP loop, by altering GTPase expression and GTP levels. It is worth mentioning that the accumulation of cGMP and PKG pathway inhibit other GTPases, like RhoA (41, 47, 48) and Rac1 (49), which may partially explain why SILD treatment also suppressed *Slfn4* expression.

In summary, we showed that the immunosuppressive properties of *Slfn4^+^* MDSCs in *Helicobacter*-induced gastric metaplasia, and ablation of these cells represents a feasible target for the blockade of SPEM development. This investigation paves the way towards a better understanding of the regulatory factors creating the host immune microenvironment after chronic infection of the stomach by *Helicobacter*. These regulatory pathways may further our development of biomarkers and therapies to prevent progression of chronic inflammation to metaplasia, a known precursor of gastric cancer.

## Data availability statement

The datasets presented in this study can be found in online repositories. The names of the repository/repositories and accession number(s) can be found below: PRJNA933232 and PRJNA933209 (SRA).

## Ethics statement

The animal study was reviewed and approved by University of Arizona.

## Author contributions

LD designed research studies, conducted experiments, analyzed data, and wrote the manuscript. SS performed cGMP ELISA analysis. RS performed animal breeding, husbandry and treatments. JM designed research studies reviewed all data, wrote and edited the manuscript. All authors contributed to the article and approved the submitted version.

## Supplementary material

Supplementary Figure 1 Heatmap of DEGs between uninfected vs infected PBMC immune cells. Representative example of immune genes enriched in the primary blood monocytes isolated from infected mice is shown. The color scale is from log2 FC-10 (blue) to log2 FC 10 (red). DEGs were identified by 10x Loupe Browser (FDR ≤ 0.05).
