# Supplementary figures and images for: Schlafen4+-MDSC in Helicobacter-induced gastric metaplasia reveals role for GTPases
Source: Front Immunol. 2023 Jun 2;14:1139391. doi: 10.3389/fimmu.2023.1139391 (PMC10272601; doi:10.3389/fimmu.2023.1139391)

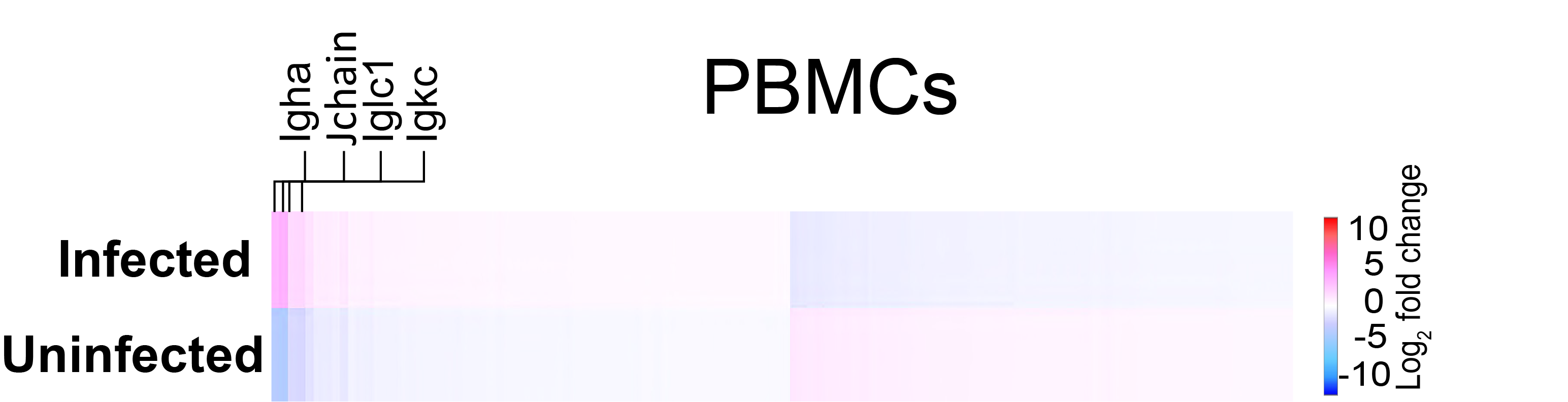

Supplement: Supplementary Figure 1 — Heatmap of DEGs between uninfected vs infected PBMC immune cells. Representative example of immune genes enriched in the primary blood monocytes isolated from infected mice is shown. The color scale is from log2 FC-10 (blue) to log2 FC 10 (red). DEGs were identified by 10x Loupe Browser (FDR ≤ 0.05). [file Image_1.tif]
