# Supplementary material for: Schlafen4+-MDSC in Helicobacter-induced gastric metaplasia reveals role for GTPases
Source: Front Immunol. 2023 Jun 2;14:1139391. doi: 10.3389/fimmu.2023.1139391 (PMC10272601; doi:10.3389/fimmu.2023.1139391)
Supplement: Supplementary file 2 [file Table_1.pdf]

Table S1. List of canonical markers used to assign the cell clusters.

Class	Figures	Cell types	Genes
PBMCs, 11 cell lineages	Fig.1	Naïve T lymphocytes	CD3e, CD4, CD8a, TCF7, SELL, LEF1, CCR7
	Fig.1	CD4+ T Helper	CD3e, CD4, GATA3, IL4, IFNG, CXCR3, CCR3, RORC2
	Fig.1	CD8+ CTL	CD3e, CD8a, GZMA, GZMB, PRF1, CTLA4, IFNG, NKG7
	Fig.1	Treg	CD4, Foxp3, IL2RA
	Fig.1	NK cells	Klrd1, NKG7, Xcl1, Klrb1b, PRF1, GZMA, NCR1
	Fig.1	NKT cells	CD4,CD8, NKG7,Xcl1, Klrb1b, PRF1, GZMA, NCR1
	Fig.1	CD14+ Mono	CD14, LYZ1,CD86, CD80, F4/80, Mafb, Chil3,
	Fig.1	DCs	CD14, CD80, CD86, CD11c, F4/80
	Fig.1	Neutrophil	CD14, CXCL2, CXCR2, Mmp9, Selp, S100a8, S100a9
	Fig.1	pDCs	PDCA-1, Siglec-H, IRF7, Ly6C,Ly6k
	Fig.1	B lymphocytes	CD19, CD79A,
Stomach CD45+, 15 cell lineages	Fig.2	Naïve CD8+ T	CD3, CD8a, Nrcam
	Fig.2	Exhausted CD8+ T	CD3, CD8a, LAG3, TIGIT, HAVCR2
	Fig.2	CD8+ CTL	CD3, CD8a, GZMA, GZMB,PRF1, CTLA4, IFNG, NKG7
	Fig.2	CD4+ Th	CD4, GATA3, IL4, IFNG, CXCR3, CCR3, RORC2
	Fig.2	Naïve CD4+T	CD4, TCF7, SELL, LEF1, CCR7
	Fig.2	γδ T	CD226, Efna5, TCRGC2, TCRGC1
	Fig.2	NKT cells	CD3e, Klrd1, NKG7,Xcl1, Klrb1b,
	Fig.2	NK cells	Klrd1, NKG7,Xcl1, Klrb1b, NCR1
	Fig.2	MALT B cells	CD19, CD79A, Jchain, IGHA
	Fig.2	Myeloid cells	CD14, CD33, Itgam, Csf3r, Csf1r,
	Fig.2	MDSCs	CD14, CD33, Itgam, NOS2, ArgI, TNF, IL1a
	Fig.2	MAST cells	KIT, Gata2, Cpa3
	Fig.2	pDCs	PDCA-1, Siglec-H, IRF7, Ly6C,Ly6k
	Fig.2	CD19+ pDC-like cell	PDCA-1, CD19, IRF7, Ly6C,Ly6k
	Fig.2	B lymphocytes	CD19, CD79A,
Subset of PBMC Slfn4+ Neutrophil	Fig.1	N1	Npg, Camp, Lft, Chil3
	Fig.1	N2	Ifit3, Gbp2, Gbp5, Gvin1, PD-L1
	Fig.1	N3	Ubox5, Map2k6,Cpeb4, ACSL1
Subset of stomach Slfn4+ cells	Fig. 2	STS4-1	Lrrc14b, Pnp2, Fam120c, Mafb, ArgI
	Fig. 2	STS4-2	Tnf, NOS2, Il1a, Il27, CXCL1,CXCL2
	Fig. 2	STS4-3	S100a8, S100a9, Trem1,Ngp, Lft, Camp
